# Progress in Medicine

**Published:** 1898-06-18

**Authors:** 


### Progress in Medicine.

### DISEASES OF THE RESPIRATORY ORGANS.

(*Continued from page* 133.)

**Asthma.**—Kingscote[1] seems to regard asthma as caused by irritation of one or more terminals of the vagus nerve, and points out that, experimentally, it can be produced only in one way, namely, by stimulating the proximal end of the divided vagus by electricity. Thus, too, it is caused in ear disease by irritation of Arnold's nerve, and in hay asthma through Meckel's ganglion. Again, in chronic heart disease there are numerous cases to be found, and the writer has noticed that many of these accompany severe dilatation of the heart, and if no other exciting cause is present they recover in proportion as the heart improves under the Schott treatment. These dilated hearts really form heavy tumours, which, he thinks, compress the vagus trunks lying behind them, and the irritation from their constant impact produces the symptoms collectively known as asthma. In proof of this, he describes ten cases where relief of the symptoms followed reduction of the size of the heart, and he suggests that benefit would arise sometimes from the use of the Schott treatment with inhalations of oxygen. In the discussion upon his paper at the Medical Society, Sansom and others were unconvinced by the physiological explanation, Sansom referring the asthma in these cases more probably to nerve irritation from the stretching which the pericardium underwent. Maguire,[2] in discussing abnormal forms of breathing, lays down that in emphysema there is deficient elasticity of the lung, which affects both expiration and inspiration, but the greater dyspnœa is inspiratory. Thus during inspiration the lower ribs sink inwards instead of moving outwards, being unsupported by proper expansion of the lungs. This in turn deprives the diaphragm of support, and leads to it only contracting feebly. The marked dyspnœa is due partly to this, but still more to the destruction of the alveolar blood vessels. In true asthma the chief difficulty is the entrance of air into the vesicles, though the dyspnœa is both expiratory and inspiratory, and the writer thinks that the congestive theory of its production is sounder than the spasmodic. Indeed, the dyspnœa of asthma only differs from that of bronchitis because of the narrower lumen of the tubes affected. Still this congestion itself is often set up by nervous influences. Cheynes Stokes' respiration seems to depend on the removal of the respiratory centre from under the control of higher cerebral ones. In yawning, sighing, or deep sleep, especially that of infants, we see irregular respiratory movements due to the same cause, and in the sleep of infants a form very like true Cheynes Stokes' breathing is not uncommon. To this loss of control from above there is added in typical cases an irritability from exhausted vitality of the respiratory centre itself.

Reveilliod[3] finds that amelioration and even cure may be obtained in some cases of asthma by injections of diphtheritic serum. He argues that the therapeutic action of drugs is exerted through the channels by which they are eliminated, and that the skin affections in asthma and after the use of serum or of iodides are so similar as to show an identical mode of elimination from the system. Hence he was led to believe that serum as well as iodides would aid in getting rid of the poison of asthma, a belief which has so far been justified by his experimental injections.

**Pneumonia.**—The production of even lobar pneumonia may be due to various organisms either singly or in conjunction, and not solely to the pneumococci of Fraenkel or Friedlander. T. W. Moore[4] describes it as a "multiple infection," caused also by the organisms peculiar to erysipelas, influenza, tuberculosis, enteric, diphtheria, anthrax, and other diseases. The inflammatory process is set up in the lung either by the organisms themselves or by the paralysing effects of their toxins. Thus, in erysipelas and influenza streptococci may be the active agents either alone or in combination with other bodies; in typhoid it is still debated whether Eberth's bacillus may enter by the lung and cause pneumonia, or whether its poison merely lays open the lung to other invaders. Besides various secondary pneumonias in phthisis, the bacillus sometimes seems to start a sudden inflammation which is called the acute tubular-pneumonic form of phthisis. Durck[5] gives a long list of the microbes found in the pneumonic lungs of children, and, moreover, discovered that a great variety even of pathogenic germs exist in the lungs of perfectly healthy animals. Finally, he shows that these germs can be injected through a tracheal tube without causing pneumonia, but if irritating dust be first injected, or the animal be suddenly chilled when warm, then the blowing in of these organisms is followed by the disease. Thus the process is easily set up by various germs when the resisting power of the lung tissue is low. Beverley Robinson[6] thinks that there is much evidence for the infective nature of broncho-pneumonia, and recommends creosote inhalations to prevent its spread. Whether we have any means for aborting it or rendering the tissues unfavourable to the growth of the invading germs, is uncertain, but the pneumococcal serum has given some measure of success. Washbourn[7] has obtained a protective serum from a pony, after nine months' injections, which has had some success, but its bactericidal powers are doubtful, and as no definite toxin has yet been isolated it is impossible to gauge its value as an anti-toxin. Centauni[8] claims to have isolated from growths of the pneumococcus certain immunising substances which have the power of uniting with certain molecules of the body to form stable chemical compounds, by which immunity is brought about. The molecules are conceived of as open chains, which are closed by union with these substances, which he calls stomoosins. Further applications of these remedies in pneumonia are to be looked for. E. Hirschfield[9] discusses the cause of the signs of a cavity which are very rarely noticed in croupous pneumonia. In his case well marked amphoric breathing occurred over a part of the right lung. There was no gangrene or abscess, no bacilli were found, and the cavernous breathing disappeared after three weeks. No satisfactory explanations of the condition have hitherto been given, but the author suggests that a circumscribed pneumothorax had been formed and limited by pleuritic adhesions. Another remarkable complication is reviewed by Sir W. Broadbent.[10] At the time of the expected crisis in a boy, though there was a little fall of temperature, a sudden disappearance of all râles and signs of consolidation took place in thirty-six hours. Now as none of the exudate was expectorated, it could only have been absorbed. Then followed muttering delirium, rapid respirations, and, before long, hyperpyrexia, all pointing to some pyæmic condition. After some progress had been made a violent paroxysmal cough pointed to enlargement of a bronchial gland. This was followed by suppuration in the mediastinum, which fortunately discharged into a bronchial tube, and recovery took place. It is not easy to explain how all symptoms of exudation from an entire lung can vanish as these did. The cases are rare and the prognosis bad. The opposite condition, delayed resolution, may be produced by various causes. Stengel[11] finds that drugs are useless, but strong counter-irritants are followed by improvement, and systemic breathing exercises are most useful. Finally the production of aseptic abscesses elsewhere by the injection of turpentine after Fochier's plan may be adopted in obstinate cases. The writer thinks the abscess acts by drawing away the leucocytes (chemotaxis) from the pulmonary area. Mr. H. Fussell,[12] in an interesting analysis of one hundred and thirty-four cases of pneumonia in a private practice with a mortality of 16·4 per cent., notices a point which is often forgotten, that the earliest signs of consolidation in a given area are dulness and diminished breath sounds, while the crepitant râle is too often absent to be reliable. De Renzi[13] advocates an antiseptic mouth wash and Pane's anti-pneumococcal serum, which has given him good results in 32 cases, since only three deaths occurred. These were otherwise almost hopeless.

[1] Lancet, April 2. [2] Clin. Jour., Jan. 10. [3] New York Med. Jour., Feb. 5. [4] Brit. Med. Jour., Jan. 1. [5] Inter. Med. Mag., Feb. [6] Med. Rec., Feb. 19. [7] Brit. Med. Jour., Dec., p. 1,849. [8] Brit. Med. Jour., Dec. 25. [9] Australas Med. Gaz., Feb. 21. [10] Brit. Med. Jour., Mar. 5. [11] Clin. Jour., Mar. 9. [12] Med. News, Mar. 5. [13] Brit. Med. Jour., April 9.

